# The battle in the apoplast: further insights into the roles of proteases and their inhibitors in plant–pathogen interactions

**DOI:** 10.3389/fpls.2015.00584

**Published:** 2015-08-03

**Authors:** Mansoor Karimi Jashni, Rahim Mehrabi, Jérôme Collemare, Carl H. Mesarich, Pierre J. G. M. de Wit

**Affiliations:** ^1^Laboratory of Phytopathology, Wageningen University and Research Centre, Wageningen, Netherlands; ^2^Department of Plant Pathology, Tarbiat Modares University, Tehran, Iran; ^3^Cereal Research Department, Seed and Plant Improvement Institute, Karaj, Iran; ^4^UMR1345, IRHS-INRA, Beaucouzé, France; ^5^Bioprotection Technologies, The New Zealand Institute for Plant and Food Research Limited, Mount Albert Research Centre, Auckland, New Zealand

**Keywords:** cysteine protease, metalloprotease, serine protease, protease inhibitor, chitinase, defence

## Abstract

Upon host penetration, fungal pathogens secrete a plethora of effectors to promote disease, including proteases that degrade plant antimicrobial proteins, and protease inhibitors (PIs) that inhibit plant proteases with antimicrobial activity. Conversely, plants secrete proteases and PIs to protect themselves against pathogens or to mediate recognition of pathogen proteases and PIs, which leads to induction of defense responses. Many examples of proteases and PIs mediating effector-triggered immunity in host plants have been reported in the literature, but little is known about their role in compromising basal defense responses induced by microbe-associated molecular patterns. Recently, several reports appeared in literature on secreted fungal proteases that modify or degrade pathogenesis-related proteins, including plant chitinases or PIs that compromise their activities. This prompted us to review the recent advances on proteases and PIs involved in fungal virulence and plant defense. Proteases and PIs from plants and their fungal pathogens play an important role in the arms race between plants and pathogens, which has resulted in co-evolutionary diversification and adaptation shaping pathogen lifestyles.

## Introduction

For successful infection of host plants and establishment of disease, fungal pathogens need weaponry to facilitate penetration, host colonization and uptake of nutrients for growth and reproduction, and at the same time to protect themselves against host defense responses. On the other hand, plants have developed surveillance systems to recognize and defend themselves against invading pathogens. Plant immune receptors recognize conserved microbe-associated molecular patterns (MAMPs) like chitin oligomers released from fungal cell walls during infection. This recognition leads to MAMP-triggered immunity (MTI) and initiates basal defense responses including the activation of structural and (bio)chemical barriers (Jones and Dangl, 2006; Spoel and Dong, 2012). However, adapted plant pathogens have gained the ability to overcome MTI by producing effector molecules that suppress or compromise MTI responses, thereby facilitating effector-triggered susceptibility (ETS; Stergiopoulos and de Wit, 2009). In response, plants have developed an additional layer of defense that enables them to recognize pathogen effectors or effector-modified host targets leading to effector-triggered immunity (ETI; Jones and Dangl, 2006).

Proteases and protease inhibitors (PIs) secreted by pathogens or their host plants have been extensively studied and have been demonstrated to play an important role in ETS and ETI (van der Hoorn, 2008). However, little is known about their role in MTI and related plant basal defense responses. Plant basal defense responses include the induction of pathogenesis-related proteins (PRs) such as antimicrobial chitinases, β-1,3-glucanases and proteases that hydrolyse the fungal cell wall components chitin, glucans, and polypeptides, respectively. The induction of these PR proteins upon plant infection, their antifungal activity, as well as their exploitation in engineering resistance in transgenic plants are very well documented (Wubben et al., 1996; Sels et al., 2008; Balasubramanian et al., 2012; Cletus et al., 2013). An early report in the literature suggested that pathogens might overcome the deleterious effects of plant chitinases by secreting proteases that modified them (Lange et al., 1996; Sela-Buurlage, 1996). This was further supported by recent studies, which indicate that chitinases are targeted by pathogen proteases and protected by PIs (Naumann et al., 2011; Slavokhotova et al., 2014). This encouraged us to review the recent advances on proteases and PIs that play a role in the arms race between plants and their fungal and oomycete pathogens.

## Plant Proteases and Protease Inhibitors Involved in Basal Defense

Most PR proteins exhibit direct antimicrobial activities, such as chitinases that degrade chitin present in fungal cell walls. PR proteins play a role in both constitutive and induced basal defense responses (Avrova et al., 2004; Shabab et al., 2008; van Esse et al., 2008). For example, tomato and potato contain basal levels of proteases in their apoplast, including serine proteases like P69, and papain-like cysteine proteases (PLCPs) like Rcr3, which are required for resistance of tomato against *Cladosporium fulvum* (Song et al., 2009), as well as Pip1 (*Phytophthora* inhibited protease 1; Tian et al., 2007; Shabab et al., 2008) and C14, which play a role in the resistance of potato against *Phytophthora infestans* (Kaschani et al., 2010; Bozkurt et al., 2011). After being challenged by pathogens, proteases are induced both locally (Tian et al., 2005) and systemically in the apoplast (Tian et al., 2007; Shabab et al., 2008; Song et al., 2009), which suggests that their activity affects pathogen growth directly or indirectly. Deletion or silencing of genes encoding these proteases enhanced the susceptibility of plants to pathogens, supporting their role in defense responses. Deletion of *Rcr3* increased the susceptibility of tomato to the late blight pathogen *P. infestans* (Song et al., 2009), to the leaf mold pathogen *C. fulvum* (Dixon et al., 2000), and also to the potato cyst nematode *Globodera rostochiensis* (Lozano-Torres et al., 2012). Likewise, silencing of *C14* in *Nicotiana benthamiana* significantly increased their susceptibility to *P. infestans* (Kaschani et al., 2010). These findings suggest that proteases have a determinative role in the execution of defense against plant pathogens.

Plant PIs have also been reported to play a role in plant immunity, through the inhibition of pathogen proteases, or the regulation of endogenous plant proteases (Ryan, 1990; Mosolov et al., 2001; Valueva and Mosolov, 2004; Kim et al., 2009). This has been shown for PIs from barley (*Hordeum vulgare*) against proteases from *Fusarium culmorum* (Pekkarinen et al., 2007), as well as for PIs from broad bean (*Vicia faba*), which inhibited the mycelial growth of several pathogens (Ye et al., 2001). The *A. thaliana* unusual serine protease inhibitor (UPI) was shown to play a role in defense against the necrotrophic fungi *Botrytis cinerea* and *Alternaria brassicicola* (Laluk and Mengiste, 2011). The UPI protein strongly inhibited the serine protease chymotrypsin but also affected the cysteine protease papain (Laluk and Mengiste, 2011). Plants harboring a loss-of-function *UPI* allele displayed enhanced susceptibility to *B. cinerea* and *A. brassicicola*, but not to the bacterium *Pseudomonas syringae*. Also, hevein-like antimicrobial peptides from wheat (WAMPs) were shown to inhibit class IV chitinase degradation by fungalysin, a metalloprotease secreted by *Fusarium verticillioides* (Slavokhotova et al., 2014). WAMPs bind to fungalysin, but are not cleaved by the enzyme due to the presence of a Ser residue between the Gly and Cys residues where cleavage of class IV chitinase by fungalysin normally takes place (Naumann et al., 2011; Slavokhotova et al., 2014). Adding equal molar quantities of WAMP and chitinase to fungalysin was sufficient to completely inhibit fungalysin activity suggesting a higher affinity of the protease to the WAMP than to the chitinase.

Interestingly, some pathogens can also manipulate the transcription of plant PIs to inhibit deleterious effects of plant proteases in their favor. For example, production of maize cysteine proteases is induced during infection by *Ustilago maydis*, but at the same time the fungus induces the production of maize cystatin CC9 that inhibits cysteine proteases to facilitate infection (van der Linde et al., 2012b; Mueller et al., 2013). This suggests an evolutionary arms race in which the infection strategy of the pathogen benefits from the host’s antimicrobial defense to suppress its defense responses.

## Fungal Proteases Targeting Host Defense Proteins

The arms race between pathogens and their hosts is often explained by recognition of MAMPs or effectors through pattern recognition receptors or resistance proteins, which results in MTI or ETI (Jones and Dangl, 2006). However, several components of basal defense are both constitutive and induced after interaction between MAMPs/effectors and immune receptors. PR proteins provide an excellent example of this. PR proteins are generally stable proteins that often exhibit a basal level of expression, but are also strongly induced after infection (Sels et al., 2008). PR proteins and their antifungal activity have been exploited to improve broad-spectrum resistance in plants. Plants such as tobacco, tomato, potato, peanut, and cacao have been engineered to over-express chitinases alone (Schickler and Chet, 1997; de las Mercedes Dana et al., 2006; Maximova et al., 2006; Iqbal et al., 2012; Cletus et al., 2013) or in combination with other PR proteins in pea and rice (Sridevi et al., 2008; Amian et al., 2011), and showed enhanced resistance to fungal pathogens.

Plant chitinases and especially chitin-binding domain (CBD)-containing chitinases play an important role in defense against pathogenic fungi (Iseli et al., 1993; Suarez et al., 2001). Some fungal pathogens such as *C. fulvum* secrete chitin-binding effector proteins like CfAvr4 into the colonized extracellular space of tomato leaves to protect themselves against the antifungal activity of apoplastic plant chitinases (van den Burg et al., 2006). Indeed, CfAvr4 binds to chitin of fungal cell walls, making chitin inaccessible to plant chitinases, thereby preventing hydrolysis by these enzymes (van den Burg et al., 2006). Functional homologs of CfAvr4 have been identified in other Dothideomycete plant pathogens, in which they likely also protect the fungal cell wall against plant chitinases (Stergiopoulos et al., 2010; de Wit et al., 2012; Mesarich et al., 2015). However, many fungal pathogens do not carry homologs of the *CfAvr4* gene in their genome. It appears that some fungi secrete proteases that cleave CBD-chitinases. For example, *F. solani* f. sp. *phaseoli* is able to modify chitinases during infection of bean to facilitate host colonization (Lange et al., 1996). Also an extracellular subtilisin protease from *F. solani* f. sp. *eumartii* was reported to modify chitinases and β-1,3-glucanases present in intercellular washing fluids of potato (Olivieri et al., 2002). More recently, it was shown that *F. verticillioides* and other maize pathogens, including *Bipolaris zeicola* and *Stenocarpella maydis*, secrete two types of proteases that truncate maize class IV CBD-chitinases (Naumann, 2011). A fungalysin metalloprotease of *F. verticillioides* was found to cleave within the CBD domain between conserved Gly and Cys residues (Naumann et al., 2011), while a novel polyglycine hydrolase present in many fungi belonging to the family of *Pleosporineae* cleaved within the polyglycine linker present in the hinge domain of class IV chitinases (Naumann et al., 2014, 2015). In another recent study it was shown that the fungal tomato pathogens *B. cinerea*, *V. dahliae*, and *F. oxysporum* f. sp. *lycopersici* secrete proteases that modify tomato CBD-chitinases (Karimi Jashni et al., 2015). For *F. oxysporum* f. sp. *lycopersici*, the synergistic action of a serine protease, FoSep1, and a metalloprotease, FoMep1 (the ortholog of fungalysin from *F. verticillioides*), was required for cleavage and removal of the CBD from two tomato CBD-chitinases (Karimi Jashni et al., 2015). Removal of the CBD from two tomato CBD-chitinases by these two enzymes led to a reduction of their chitinase and antifungal activity. In addition, mutants of *F. oxysporum* f. sp. *lycopersici* lacking both *FoSep1* and *FoMep1* exhibited reduced virulence on tomato, confirming that secreted fungal proteases are important virulence factors by targeting CDB-chitinases to compromise an important component of plant basal defense (Karimi Jashni et al., 2015).

Collectively, the activity of fungal proteases might explain why overexpression of plant chitinases in transgenic plants has not become an effective strategy to obtain durable resistance against fungal pathogens. Secretion of proteases and PIs by pathogens to modify, degrade, or inhibit basal defense proteins might have played an important role during co-evolution with their host plants (Hörger and van der Hoorn, 2013). Therefore, overexpression of chitinases from a heterologous source in transgenic plants might be a more efficient approach to obtain durable resistance against pathogens, as they have not co-evolved with these “foreign” defense proteins.

## Fungal Protease Inhibitors Targeting Host Proteases

**FIGURE 1:**
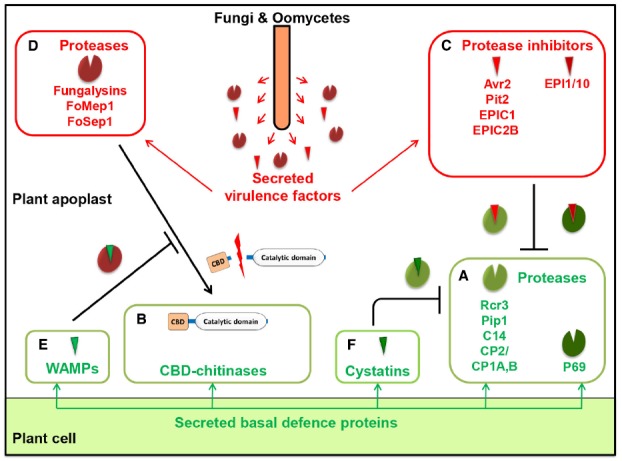
**Proteases and protease inhibitors at the plant–pathogen interface.** As part of their basal defense response, plants secrete deleterious enzymes such as proteases **(A)** and chitin-binding domain (CBD)-containing chitinases **(B)** that target pathogen components. In response, filamentous pathogens secrete protease inhibitors **(C)** that inhibit plant cysteine or serine proteases. Filamentous pathogens also secrete fungalysin metallo- or serine proteases **(D)** that process antifungal CBD-chitinases of plants. In response, plants secrete antimicrobial peptides such as hevein-like antimicrobial peptides from wheat (WAMPs) **(E)** that inhibit fungalysin metalloproteases or cystatins **(F)** that inhibit endogenous plant cysteine proteases. Examples shown in this figure are discussed in the text.

Plant pathogens also secrete PI effectors to inhibit plant defense proteases and promote disease development. These effectors are targeted to various host compartments (Tian et al., 2009). One such effector, Avr2, secreted by *C. fulvum* during infection, is required for full virulence of this fungus on tomato (Rooney et al., 2005). Avr2 inhibits the tomato apoplastic PLCPs Rcr3 and Pip1 to support growth of *C. fulvum* in the apoplast. Also, plants expressing Avr2 showed increased susceptibility to other pathogenic fungi, including *B. cinerea* and *V. dahliae* (van Esse et al., 2008). Moreover, *A. thaliana* plants expressing Avr2 triggered global transcriptional reprogramming, reflecting a typical host response to pathogen attack (van Esse et al., 2008). Two other PI effectors are the cystatin-like proteins EPIC1 (extracellular proteinase inhibitor C1) and EPIC2B (extracellular proteinase inhibitor C2B), whose expression is strongly induced in the oomycete *P. infestans* during biotrophic growth on tomato leaves (Tian et al., 2007; Song et al., 2009). These PIs selectively target the plant PLCPs Rcr3, Pip1, and C14 in the apoplast of potato and tomato. The EPICs inhibit C14 and possibly other PLCPs over a wider pH range than that observed for Avr2, which only inhibits Pip1 and Rcr3 at pH values occurring in the apoplast where the pathogen grows. In addition, *P. infestans* secretes two serine PIs (EPI1 and EPI10) that target and inhibit the major apoplastic serine protease P69B, likely to decrease its role in defense (Tian et al., 2004, 2005). It was proposed that EPI1 protects EPIC1 and EPIC2B proteins from degradation by P69B (Tian, 2005). Furthermore, the maize pathogen *U. maydis* secretes the cysteine PI Pit2 that strongly inhibits three abundant defense-related maize cysteine proteases (CP2 and its two isoforms CP1A and CP1B; Van der Linde et al., 2012a; van der Linde et al., 2012b; Mueller et al., 2013). These findings indicate that cysteine and serine PIs secreted by different groups of filamentous fungal and oomycete pathogens, as well as their activity against plant proteases, can compromise plant basal defense responses. A schematic overview of different types of interactions between pathogen and host proteases and PIs at the plant–pathogen interface is presented in Figure 1.

## Proteases, PI Effectors, and Their Role in Receptor-Mediated Host Defense Responses

The plant immune system is able to recognize pathogen effectors to mount receptor-mediated defense responses. Although the intrinsic function of protease and PI effectors secreted by some pathogenic fungi promote disease through manipulation of host defense, proteases and PI effectors can also be recognized by host immune receptors mediating defense responses. This adaptation and counter-adaptation reflects the arms race between pathogens and their host plants. A clear example of such an evolutionary arms race are the cysteine PIs Avr2 from *C. fulvum* and Gr-VAP1 (*Globodera rostochiensis* Venom Allergen-like Protein) from *G. rostochiensis* that bind and inhibit the tomato cysteine protease Rcr3^pim^. The tomato immune receptor protein Cf-2 senses this interaction and mediates the induction of defense responses (Song et al., 2009; Lozano-Torres et al., 2012). Most likely, the interaction causes a conformational change in Rcr3, which is recognized by the Cf-2 receptor (Krüger et al., 2002; Rooney et al., 2005). This hypothesis is supported by the finding that a natural variant of Rcr3 is recognized by Cf-2 in an Avr2-independent manner (Dixon et al., 2000). Moreover, in tomato plants lacking the Cf-2 receptor, targeting of Rcr3 is not sensed and plants are more susceptible to *G. rostochiensis* (Lozano-Torres et al., 2012).

## Co-evolution Between Plants and Their Pathogens is Reflected by the Numerous Variant Proteases and PIs in the Genomes of Both Organisms

The genomes of fungal plant pathogens encode predicted proteases belonging to various subfamilies that vary in number between pathogens with different lifestyles. Generally, hemi-biotrophs and saprotrophs contain higher numbers of secreted proteases than biotrophs (Ohm et al., 2012). However, these predictions are based on gene numbers and may not be supported by their transcription and translation profiles. For example, *C. fulvum*, which is a biotrophic fungus, has numbers of proteases that are comparable to the phylogenetically closely related hemi-biotroph *Dothistroma septosporum* (de Wit et al., 2012). However, likely due to its adaptation to a different host and lifestyle, many *C. fulvum* protease genes are not expressed *in planta* and some have undergone pseudogenization (van der Burgt et al., 2014). Deletion and duplication of protease genes were reported to occur in the genome of the grass endophytic fungus Epichloë festucae (Bryant et al., 2009) but their biological implications have not yet been studied.

Adaptation of PI effectors from pathogens to inhibit different host proteases has been observed in several cases. The Avr2 PI of *C. fulvum*, for example, has a high affinity for the host proteases Rcr3 and Pip1 and a low affinity for C14 (Shabab et al., 2008; Hörger et al., 2012). *P. infestans* EPICs have a high affinity for C14 and a low affinity for Rcr3 and Pip1 (Kaschani et al., 2010). Furthermore, *U. maydis* Pit2 inhibits the maize cysteine proteases CP1, CP2, and XCP2, but does not inhibit cathepsin CatB (Mueller et al., 2013). Different types of selection pressure may lead to the circumvention of protease inhibition by PIs. For example, purifying or diversifying selection has been reported for the proteases Rcr3, C14, and Pip1, and has been shown to act at their PI binding sites. Sequencing of the tomato proteases Rcr3 and Pip1 across different wild tomato species has shown that these proteins are under strong diversifying selection imposed by Avr2. For instance, one of the variant residues in the binding site of Rcr3 prevented inhibition by Avr2, indicating selection for evasion from recognition by this inhibitor (Shabab et al., 2008). C14 from solanaceous plants is also the target of EPICs secreted by *P. infestans* and is under diversifying selection in potato and under conservative selection in tomato. This demonstrates that C14 plays an active role in host immunity against this pathogen and variations in the sequence of C14 in natural hosts of *P. infestans* highlight the co-evolutionary arms race at the plant–pathogen interface (Kaschani et al., 2010).

Evolutionary diversification may vary from point mutation to gene deletion or insertion. EPIC1 and EPIC2 are PIs present in *P. infestans*, however their orthologs were lost in *P. sojae* and *P. ramorum* (Tian et al., 2007). *P. mirabilis*, a species closely related to *P. infestans*, is a pathogen of *Mirabilis jalapa*, and secretes the PI PmEPIC1, an ortholog of EPIC1 that inhibits C14 but not Rcr3 (Dong et al., 2014). However, *M. jalapa* secretes MRP2, a PLCP homolog of Rcr3, that is more effectively inhibited by PmEPIC1 than by EPIC1 (Dong et al., 2014). Substitution of one amino acid residue in PmEPIC1 and EPIC1 restored the inhibitory function of PmEPIC1 on Rcr3 and of EPIC1 on MRP2, respectively. These results show that proteases and PIs have played important roles in adaptation of the two *Phytophthora* species to their respective host plants, although the two species diverged only a 1000 years ago (Dong et al., 2014). This is an excellent example for a role of a protease and PI in the arms race between a plant and its pathogen and exemplifies how diversification and adaptation of a protease-PI complex may work at the molecular level.

## Conclusion and Perspective

The recent advances reviewed here exemplify determinative roles of proteases and PIs in shaping plant–pathogen interactions. Analyses of genome databases of both plants and pathogens show that these organisms encode numerous proteases and PIs, of which we are just beginning to understand some of their roles. Advanced transcriptome and proteome tools such as RNA sequencing and protease profiling will facilitate identification of important proteases and PIs for further functional analysis. The redundancy of proteases in pathogens is a technical challenge that has so far hampered defining their biological functions. Targeted deletion of one or even two protease genes failed to change virulence of the plant pathogenic fungi *Glomerella cingulata* (Plummer et al., 2004) and *B. cinerea* (ten Have et al., 2010), respectively. Karimi Jashni et al. (2015) could only show decreased virulence of a double protease mutant of the tomato pathogen *F. oxysporum* by a combined biochemical and genetic approach, and using a defined plant enzyme (CBD-chitinase) as a substrate that was presumed to be involved in plant defense. This indicates that multi-gene targeting of protease and PI genes to identify their role in virulence or avirulence remains a challenge in filamentous fungi. Targeting multiple protease and PI genes might also be hampered by lack of sufficient numbers of selection markers for targeted gene replacement. In the latter case multiple protease and PI genes might be targeted by targeted gene silencing.

## Author Contributions

MJ and PdW conceived and wrote the review; JC, RM, and CM critically reviewed the manuscript.

### Conflict of Interest Statement

The authors declare that the research was conducted in the absence of any commercial or financial relationships that could be construed as a potential conflict of interest.
